# Preparation of Zn−Gly and Se−Gly and Their Effects on the Nutritional Quality of Tea (*Camellia sinensis*)

**DOI:** 10.3390/plants12051049

**Published:** 2023-02-25

**Authors:** Feixia Li, Xinzhuan Yao, Litang Lu, Yujie Jiao

**Affiliations:** 1 The Key Laboratory of Plant Resources Conservation and Germplasm Innovation in Mountainous Region (Ministry of Education), College of Life Science, Guizhou University, Guiyang 550025, China; 2 College of Tea Science, Institute of Plant Health & Medicine, Guizhou University, Guiyang 550025, China

**Keywords:** Zn−Gly, Se−Gly, Se nutrition biofortification, Zn nutrition biofortification, tea plants, aminochelates

## Abstract

Background: Micronutrient malnutrition affects millions of people due to a lack of Zn and Se. Methods: The process conditions for the manufacture of glycine−chelated sodium selenite (Se−Gly) and zinc sulfate heptahydrate (Zn−Gly) were studied. The effects of ligand concentration, pH, reaction ratio, reaction temperature, and reaction time on fertilizer stability were assessed. The effects of Zn−Gly and Se−Gly on tea plants were determined. Results: Orthogonal experiments showed that the optimal preparation conditions for Zn−Gly (75.80 % Zn chelation rate) were pH 6.0, ligand concentration 4 %, reaction ratio 1:2, reaction time 120 min, reaction temperature 70 ℃. The optimal preparation conditions for Se−Gly (56.75 % Se chelation rate) were pH 6.0, ligand concentration 10%, reaction ratio 2:1, reaction time 40 min, temperature 50 ℃. Each chelate was completely soluble in water and verified by infrared spectroscopy and ultraviolet spectroscopy. Conclusions: Zn−Gly and Se−Gly increased the Zn and Se content in tea plants, and foliar application was more effective than soil application. Combined application of Zn−Gly and Se−Gly was more effective than Zn−Gly or Se−Gly alone. Our findings suggest that Zn−Gly and Se−Gly provide a convenient method of addressing human Zn and Se deficiency.

## 1. Introduction

Micronutrient imbalance in the human diet is a global nutritional problem [1], and zinc and selenium are the elements most associated with micronutrient malnutrition [2]. Zn and Se are essential trace elements for human growth and development [3]. As per recent reports, Zn and Se might even facilitate the fight against the unprecedented global burden of COVID-19 infection [4,5,6,7,8]. Despite the importance of these micronutrients, more than 30% of the world’s population is Zn−deficient [9], while 15% of the global population is Se−deficient, with these deficiencies occurring in both developed and developing countries [10]. This relatively common deficiency has become a limiting factor for crop productivity in global agricultural land [11], and mineral malnutrition has a considerable negative impact on individual health [12]. This widespread phenomenon poses a threat to human health and is known as “hidden hunger” [13].

In the battle against global micronutrient malnutrition, agronomic approaches focusing on fertilization have been shown to be effective methods of enhancing Zn and Se levels in food crops [2,14,15]. Ideal fertilization strategies concentrate micronutrients in the edible parts of crops without affecting yield, and such methods represent the most economically viable ways to alleviate micronutrient malnutrition worldwide. Zinc sulfate heptahydrate and sodium selenite are the forms of inorganic Zn and Se that are most commonly used to improve Zn and Se deficiency [16,17]. However, both of these compounds have low efficiency and great potential for environmental pollution, so they are not ideal for human dietary applications [18]. As an innovative type of organic fertilizer, chelated trace elements are not readily fixed in soil and are soluble in water. In addition, chelated trace elements can be mixed with other solid or liquid fertilizers without chemical reaction or reduction of any fertilizer [19,20]. The application of conventional fertilizers and commercially available synthetic chelating agents such as EDTA is unsustainable, but amino chelates are safer and more effective forms of fertilizer that have better plant biofortification performance and reduced environmental risk, and they are thus considered to be suitable replacement candidates [21]. Following the application of an amino acid−chelated fertilizer to the leaf surface, the compound is not easily dissociated, allowing it to be efficiently absorbed and utilized by plants. Other important advantages of amino acid−chelated fertilizers in comparison with conventional fertilizers are their lower photodegradation rates, reduced toxicity, better metal ion binding capacity, and superior plant growth−promoting effects [22]. However, many fertilizer products sold around the world that claim to be amino acid chelates may simply be mixtures or recombined substances that are not true chelates [21].

Tea (*Camellia sinensis* L.), as a zinc−rich and selenium−rich plant, is a beneficial crop for Zn and Se biofortification. It has become one of the most popular plants in the human diet because of its beneficial effects on the human body [23]. As a functional food, tea plants can fill the nutritional gaps of Zn and Se that primary staple crops cannot. In addition, polyphenols contained in tea are considered to be immune−enhancing nutrients, which can effectively regulate the body’s innate immune response and have been shown to enhance the immune response to the novel coronavirus disease COVID-19 [24]. Therefore, in order to optimize the health benefits of *C. sinensis*, various types of biofortified functional tea have been developed [25]. The application of amino acid−chelated micronutrients, such as glycine−chelated Zn (Zn−Gly) and Se (Se−Gly) fertilizers, seems to represent an ideal method of achieving efficient and effective biofortification of tea plants. However, the optimal process conditions for the production of Zn−Gly and Se−Gly have not been reported.

In this study, the optimum process conditions for the manufacture of Zn−Gly and Se−Gly were studied at laboratory scale using an orthogonal experimental design, with the goal of improving the economic value of tea, tea quality, and the effectiveness of Zn and Se nutrition for people with Zn and Se deficiency. The effects of ligand concentration, mass ratio of ligand to reactant, pH value, chelation time and temperature were assessed. Each chelate was verified by infrared spectroscopy and ultraviolet spectroscopy. The combination of process parameters that produced the highest content of Zn and Se in the resulting Zn−Gly and Se−Gly was selected for pot experiments with tea plants to explore the effects of these chelates alone and in combination.

## 2. Results

### 2.1. Determination of the Chelation Rate of Zn and Se Chelated by Gly

Previous research showed that ligand concentration, pH, reaction ratio, reaction temperature, reaction time, and other parameters have an impact on chelate yield [26,27]. An orthogonal table (L_16_ (4^5^)) was used to investigate these five factors at four levels in orthogonal experiments, with three replicates for each treatment. The experimental results are shown in Table 1 and Table 2. According to the R values, the order of influence of various factors on the chelation rate of zinc was as follows: A (pH) > B (ligand concentration) > C (reaction ratio) > D (reaction time) > E (reaction temperature). The order of influence of the factors on the chelation of selenium was found to be consistent with that of zinc. The optimal process conditions for the preparation of Zn−Gly (zinc chelation rate 75.80 %) were A_2_B_2_C_1_D_4_E_3_ (pH 6.0, ligand concentration 4 %, ratio of glycine to zinc sulfate 1:2, reaction time 120 min, reaction temperature 70 °C). The optimal process conditions for the preparation of Se−Gly (selenium chelation rate 56.75 %) were A_2_B_4_C_3_D_2_E_1_ (pH 6.0, ligand concentration 10 %, ratio of glycine to sodium selenite 2:1, reaction time 40 min, reaction temperature 50 °C). Zn−Gly and Se−Gly produced under the conditions described above were relatively stable.

### 2.2. Characterization of Zn and Se−Gly Chelates

Zn−Gly and Se−Gly were produced using the optimal conditions according to the orthogonal test results, and their physical characteristics and molecular structure were analyzed. The physical characteristics of each chelate are shown in Table 3.

**Figure 1:**
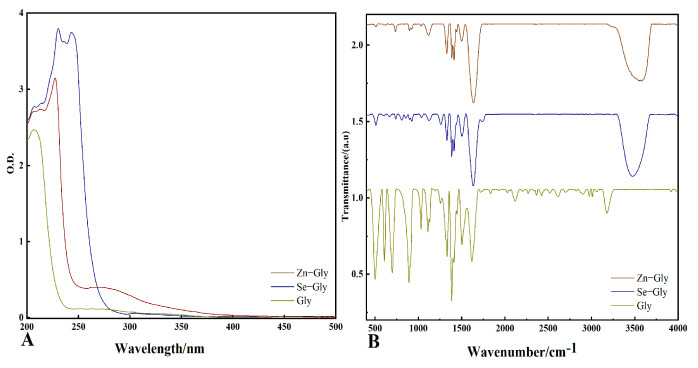
(**A**) UV scanning analysis of glycine−chelated zinc (Zn−Gly), glycine−chelated selenium (Se−Gly), and the standard glycine (Gly); (**B**) FT−IR spectra of Zn−Gly, Se−Gly, and the standard glycine (Gly).

The structure of each chelate was analyzed by UV spectrophotometry. Differences in samples after chelation can be analyzed based on changes in the dislocation and strength of UV absorption spectra [28]. As shown in Figure 1A, purified water was used as a blank, and a baseline was established at 200–500 nm. The obtained diluents were scanned at 200–500 nm, and the absorbance of Gly was significantly lower than that of Zn−Gly and Se−Gly. The main absorption peak of Gly was at 206.5 nm, and the absorbance was 2.46. The main absorption peak of Zn−Gly was at 228 nm, and the absorbance was 3.13. The main absorption peak of Se−Gly was located at 231.8 nm, and the absorbance was 3.78. Functional groups were analyzed by FTIR spectroscopy [29]. The infrared spectra of Gly, Zn−Gly, and Se−Gly in the wavenumber range of 400–4000 cm^−1^ are shown in Figure 1B. The formation of an NH_2_−M bond and a COO-M bond and the disappearance of an NH_3_-glycine bond and a COO- bond indicated the formation of a membered ring structure by the chelated glycinates. Gly was present in the crystalline state in the form of zwitterions, with charges contributed primarily by the main functional groups of NH_3_, NH−, and COO-. After Gly formed a chelate with zinc sulfate heptahydrate or sodium selenite, some of its main absorption peaks shifted significantly, which, along with changes in their relative intensity, confirmed the formation of new substances. The relative intensity of the hydroxyl association peak was reduced because the influence of other groups increased after the coordination reaction, and the peak at about 3100 cm^−1^ was shifted. The absorption peak at 1408 cm^−1^ was attributed to a -CH- group. The −NH_3_^+^ characteristic peak of glycine at about 2000 cm^−1^ was absent from the infrared spectrum of the chelate. The characteristic absorption peaks of N−H stretching vibration appeared at 3326 cm^−1^ and 3262 cm^−1^. The absorption peaks at 1599 cm^−1^ and 1392 cm^−1^, which were attributed to the stretching vibration of COO-, moved to a lower wavenumber and higher wavenumber, respectively, in comparison with the ligand, indicating that COO- was monodentate and demonstrating the presence of a chelating ring in the molecule. The R-N stretching vibration peaks at 543 cm^−1^ and 456 cm^−1^ further illustrate the formation of the chelate. A broad absorption peak appeared near 3400 cm^−1^, indicating the presence of water molecules in the chelate. Since only one oxygen atom in the carboxylate ion was involved in coordination, water molecules likely formed hydrogen bonds between the two chelate molecules. The change in the characteristic absorption peak showed that the chelated formed by the reaction of glycine with zinc or selenium ions were new compounds that differed from the parent molecule. The carbonyl and amino groups in the carboxyl groups on the side chain or end group were determined to be in the Gly molecule.

### 2.3. Effects of Zn−Gly and Se−Gly on the Chlorophyll Content of Tea Plants

**Figure 2:**
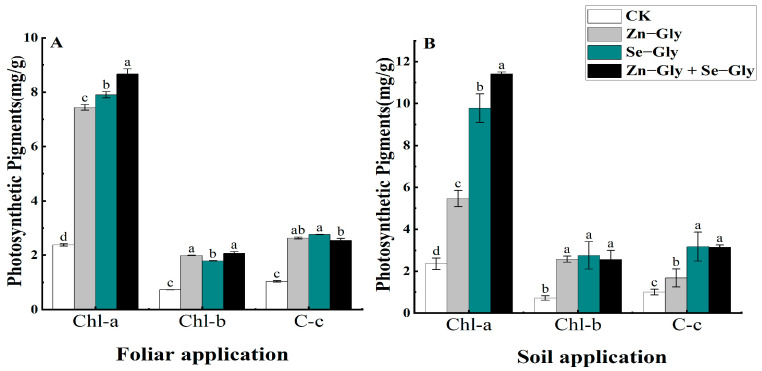
Effects of different chelated fertilizers on the chlorophyll content of tea leaves under different application methods. (**A**) Changes in the chlorophyll content in tea leaves under different foliar fertilizers. (**B**) Changes in the chlorophyll content in tea leaves under different soil fertilizers. The abscissa represents the different photosynthetic pigments in tea, in order: chlorophyll a (Chl−a), chlorophyll b (Chl−b) and carotenoids (C−c). Different colors represent different treatments, from left to right: CK, Zn−Gly, Se−Gly, Zn−Gly + Se−Gly. Results labeled with the same letters on the histogram are not significantly different at *p* < 0.05.

In order to study the effects of different Zn and Se chelate treatments on the growth of tea plants, experiments were performed to measure the chlorophyll content of tea leaves after 10 days of Zn−Gly and/or Se−Gly treatment via foliar and soil application. The concentrations of chlorophyll a, chlorophyll b, and carotenoids in tea leaves following each treatment reflect the intensity of plant photosynthesis, as shown in Figure 2. Foliar and soil application of Zn−Gly, Se−Gly, or the combination of Zn−Gly + Se−Gly promoted the production of chlorophyll and carotenoids (Figure 2A,B). In comparison with the control treatment, the combined application of Zn−Gly + Se−Gly significantly promoted photosynthesis, in agreement with a previous report [30].

### 2.4. Effects of Zn−Gly and Se−Gly on the Total Se and Total Zn Content in Tea

#### 2.4.1. Effects of Different Treatments on the Zn and Se Content in Tea

**Figure 3:**
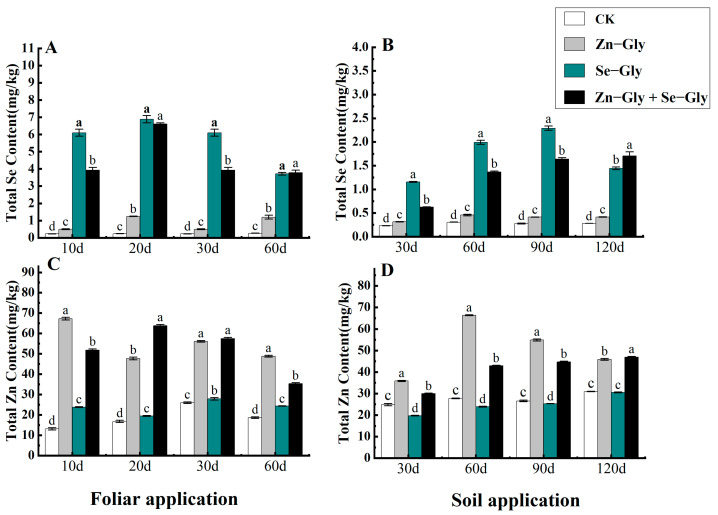
The content of zinc and selenium in tea leaves under different fertilization treatments. (**A**,**C**) Content of zinc and selenium in tea leaves following foliar fertilizer application; (**B**,**D**) Content of zinc and selenium in tea leaves following soil fertilizer application. The abscissa shows the days of treatment. Different colors represent different treatments, from left to right: CK, Zn−Gly, Se−Gly, Zn−Gly +Se−Gly. Results labeled with the same letters on the histogram are not significantly different at *p* < 0.05.

The effects of foliar and soil application of Zn−Gly, Se−Gly and Zn−Gly + Se−Gly on the Zn and Se content in tea are shown in Figure 3. Foliar and soil application of Se−Gly and Zn−Gly + Se−Gly significantly affected the Se content of tea leaves in comparison with the control (CK) treatment (*p* < 0.05). The order of the effects of the treatments on Se content was Se−Gly > Zn−Gly + Gly−Se > Zn−Gly > CK as shown Figure 3A,B. Foliar and soil application of Zn−Gly also increased the Se content in comparison with the CK treatment. The Se content in tea leaves was 1.16–6.88 mg/kg under foliar and soil Se−Gly treatment, which was significantly higher than the content following the CK treatment (*p* < 0.05). The Se content in tea leaves was 0.63–6.60 mg/kg under foliar and soil Zn−Gly + Se−Gly treatment, which was significantly higher than the content following the CK treatment (*p* < 0.05).

Foliar and soil application of Zn−Gly and Zn−Gly + Se−Gly significantly affected the Zn content of tea leaves (*p* < 0.05). Foliar application of Se−Gly increased the Zn content of tea leaves, and no regular trend was observed in the change of Zn content in soil. The order of the effects of the treatments on Zn content was Zn−Gly > Zn−Gly + Se−Gly > Se−Gly as shown in Figure 3C,D. Foliar and soil application of Zn−Gly significantly increased the Zn content of tea leaves (35.84–67.22 mg/kg) in comparison with that observed following the CK treatment (*p* < 0.05). Foliar and soil application of Zn−Gly +Se−Gly significantly increased the Zn content of tea leaves (30.00–63.80 mg/kg) in comparison with that observed following the CK treatment (*p* < 0.05).

#### 2.4.2. Changes in the Zn and Se Content in Tea Leaves at Different Picking Times

**Figure 4:**
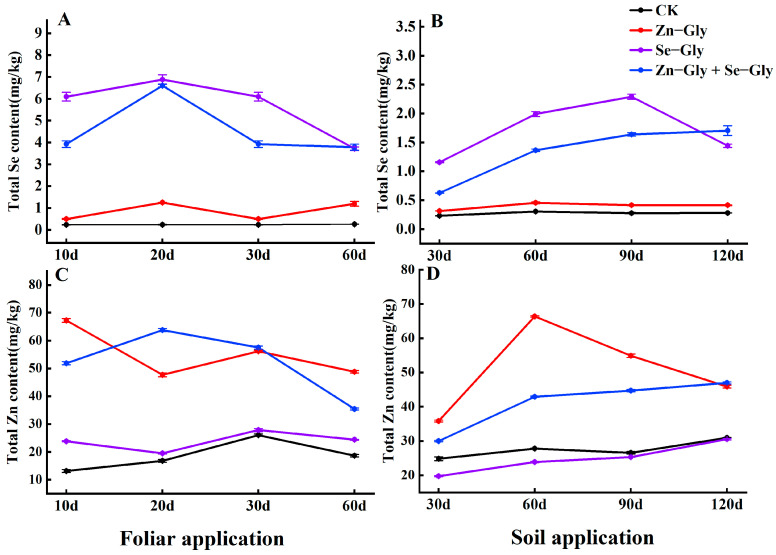
The content of zinc and selenium in tea leaves under different treatments. (**A**,**C**) Content of zinc and selenium in tea leaves following foliar fertilizer application; (**B**,**D**) Content of zinc and selenium in tea leaves following soil fertilizer application. The abscissa shows the days of treatment. Different colors represent different treatments, as follows: black, CK; red, Zn−Gly; purple, Se−Gly; blue, Zn−Gly + Se−Gly.

Tea plants absorb Zn−Gly and Se−Gly very quickly after they are sprayed on the leaves. After Se−Gly and Zn−Gly + Se−Gly treatment, the Se content of tea leaves first increased and then decreased. Following Se−Gly treatment, the maximum Se content (6.89 mg/kg) was observed on the 20th day (Figure 4A). Following Zn−Gly + Se−Gly treatment, the maximum Se content (6.60 mg/kg) was observed the 20th day (Figure 4A). Following treatment with Zn−Gly alone, maximum Zn content (67.22 mg/kg) was reached on the 10th day (Figure 4C), and this increase was slower than that observed following the CK treatment. Following combined application of Zn−Gly + Se−Gly, the Zn content of tea leaves first increased and then decreased, and the maximum Zn content (63.80 mg/kg) was observed on the 20th day (Figure 4C).

Following soil application of the chelates, the Se content of tea leaves first increased and then decreased slowly. Following Se−Gly treatment, the maximum Se content (2.29 mg/kg) was observed at 90 days (Figure 4B). Following treatment with Zn−Gly + Se−Gly, Se content showed a slow upward trend, and maximum Se content (1.70 mg/kg) was observed at 120 days (Figure 4B). Following treatment with Zn−Gly alone, the Zn content (66.37 mg/kg) was observed at 60 days (Figure 4D), after which it showed a downward trend. The combined application of Zn−Gly + Se−Gly produced a gradual upward trend in the Zn content of tea leaves throughout the experiment, and the maximum Zn content (47.00 mg/kg) was observed at 120 days (Figure 4D).

### 2.5. Effects of Zn−Gly and Se−Gly on Nutrient Components of Tea in Different Picking Stages

#### 2.5.1. Free Amino Acids (AA)

**Figure 5:**
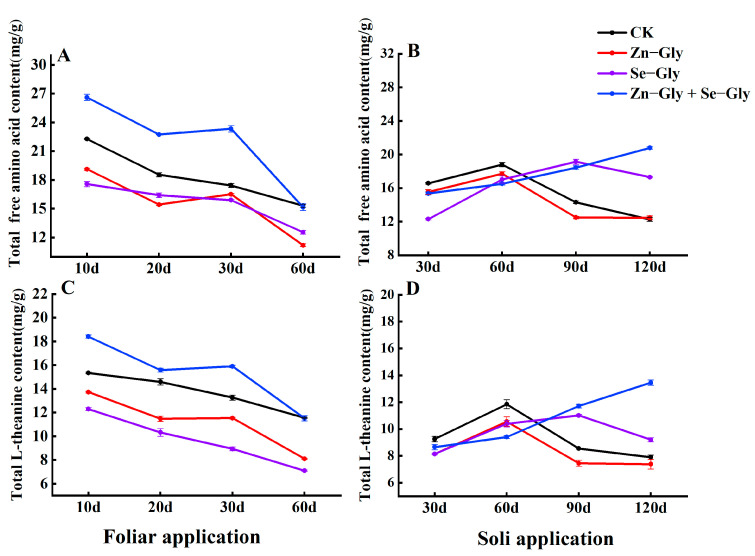
The content of total free amino acids and L−theanine in tea leaves under different fertilization treatments. (**A**,**C**) Content of total free amino acids and L−theanine in tea leaves following foliar fertilizer application; (**B**,**D**) Content of total free amino acids and L−theanine in tea leaves following soil fertilizer application. The abscissa shows the days of treatment. Different colors represent different treatments, as follows: black, CK; red, Zn−Gly; purple, Se−Gly; blue, Zn−Gly + Se−Gly.

Theanine is the predominant free AA in tea. As shown in Figure 5, foliar application of Zn−Gly or Se−Gly alone significantly reduced the content of AA in comparison with the CK treatment (*p* < 0.05). Soil or foliar application of the combination of Zn−Gly + Se−Gly significantly increased the content of AA in comparison with the CK treatment as shown Figure 5A,B (*p* < 0.05). The L−Theanine content measured following each fertilizer treatment was consistent with the corresponding AA measurement.

#### 2.5.2. Tea Polyphenol (TPs)

**Figure 6:**
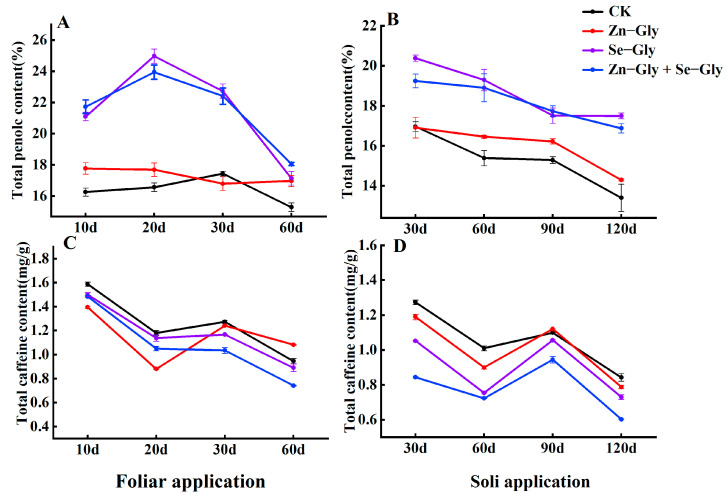
The content of tea polyphenols and caffeine in tea leaves under different treatments. (**A**,**C**) Content of tea polyphenols and caffeine in tea leaves following foliar fertilizer application; (**B**,**D**) Content of tea polyphenols and caffeine in tea leaves following fertilizer soil application. The abscissa shows the days of treatment. Different colors represent different treatments, as follows: black, CK; red, Zn−Gly; purple, Se−Gly; blue, Zn−Gly + Se−Gly.

Foliar and soil application of Zn−Gly alone, Se−Gly alone, and Zn−Gly + Se−Gly increased the TP content of tea leaves in comparison with the CK treatment (Figure 6A,B) (*p* < 0.05). Following foliar fertilizer application, the TP content of tea leaves first increased and then decreased, which was consistent with the changes in Zn and Se content. Following soil fertilizer application, the TP content of tea leaves showed a trend of first increasing and then stabilizing, but the TP content observed after foliar and soil application of each chelate treatment was significantly higher than that observed following the CK (*p* < 0.05) (Figure 6A,B).

#### 2.5.3. Caffeine (CAF)

CAF is an important bioactive component of tea that plays a key role in determining tea quality. Foliar and soil application of Se−Gly alone and Zn−Gly + Se−Gly reduced the CAF content in comparison with the CK treatment (*p* < 0.05) (Figure 6C,D). Following the foliar and soil application of Zn−Gly alone, the CAF content of tea leaves showed a trend of first decreasing, then increasing, then finally decreasing again.

#### 2.5.4. Catechins

**Figure 7:**
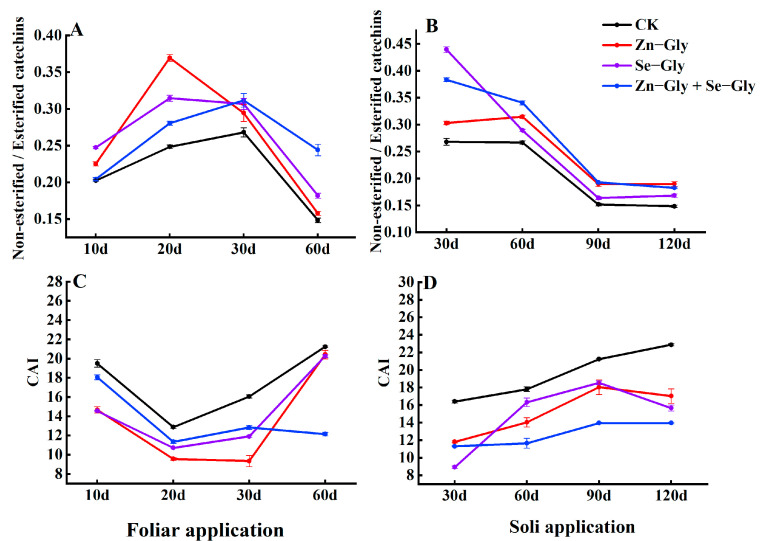
The non−esterified/esterified catechin index and catechin bitterness index in tea leaves under different fertilization treatments. (**A**,**C**) Non−esterified/esterified catechin index and catechin bitterness index in tea leaves following foliar fertilizer application; (**B**,**D**) Non−esterified/esterified catechin index and catechin bitterness index in tea leaves following soil fertilizer application. The abscissa shows the days of treatment. Different colors represent different treatments, as follows: black, CK; red, Zn−Gly; purple, Se−Gly; blue, Zn−Gly + Se−Gly.

There are many kinds of catechins, which can be roughly divided into ester catechins and non−ester catechins. Following soil and foliar application of Zn−Gly alone, Se−Gly alone, and Zn−Gly + Se−Gly, the change in the catechin quality index was consistent with the changes in the Zn and Se content of tea leaves (Figure 7). However, the bitterness and astringency of each treatment at different time points were lower than those measured following the CK treatment (*p* < 0.05). In addition, the non−esterified/esterified index measured following each chelate treatment was higher than that measured following the CK treatment (*p* < 0.05)

## 3. Discussion

### 3.1. Glycine−Chelated Zinc and Selenium

The development of amino−chelated agents represents significant progress in plant nutrition [21]. In comparison with conventional fertilizers or other commercial synthetic chelating agents such as EDTA, amino chelates represent a safer and more efficient form of fertilizer, leading to better plant performance and less environmental risk [21]. In a recent study, zinc and selenium were most associated with global micronutrient malnutrition [2], and both of these are essential trace elements for human growth and development [3]. Therefore, researchers around the world continue their attempts to develop Se−and Zn−enriched food products to minimize their related deficiency disorders [31].

Amino chelates were first developed as Fe chelates to improve the iron concentration in biological systems [32,33,34]. Chelated Fe had a significant and positive influence on most quantitative and qualitative fruit properties [30,35]. In a recent study, the synthesis of organic zinc chelates with small amounts of amino acids was assessed as a method of improving tomato yield and zinc nutrition [36]. Several studies have shown that aminochelates are absorbed and utilized more efficiently compared to simple salts such as chlorides and sulfates [35,37,38]. Therefore, creating amino acid−chelated forms of zinc and selenium fertilizer to improve plant growth and nutritional quality components is critical.

The chelation rate is an important indicator of the quality of chelating fertilizers, as well as a quantitative index used to measure the degree of reaction [39]. In this study, the chelated products Zn−Gly and Se−Gly were successfully obtained. The zinc chelation rate was 75.80%, whereas the selenium chelation rate was 56.75 %. Zn−Gly and Se−Gly both showed high stability and complete solubility in water. The ultraviolet spectra of Zn−Gly and Se−Gly were red shifted to different degrees in the range 200−500 nm in comparison with that of glycine. The FT−IR spectra of Zn−Gly and Se−Gly in the range 400–4000 cm^−1^ showed that their functional groups had been changed by the process of chelation.

### 3.2. Effects of Zn−Gly and Se−Gly on Zinc and Selenium Content in Tea Plants

The results of the pot experiments showed that the contents of Se and Zn in tea leaves were about 0.19 and 13.47 mg/kg, respectively, in the absence of exogenous Zn and Se. Considering that the human demand for Zn and Se is about 15 mg Zn day^−1^ and 55 µg Se day^−1^, respectively [40], the content of Se and Zn in tea leaves alone is insufficient to meet the physiological needs of humans. Therefore, the implementation of strategies such as agronomic biofortification can be used to enhance Zn and Se content to help alleviate deficiency in these micronutrients. In general, commonly used chemical salts with high reactivity and low efficiency are not effective means of correcting micronutrient deficiencies [41,42]. Moreover, excessive application of chemical salts may cause adverse effects such as leaf burns, growth inhibition, metal element imbalance, and yield loss at harvest [43,44]. Therefore, in many cases, chelated micronutrients are the most suitable fertilizer for meeting plant nutrient requirements and provide healthy growth and high yield outcomes [45]. Application of amino chelates instead of simple conventional fertilizers generally results in higher nutrient uptake efficiency and fewer negative side effects [45,46,47].

In this study, glycine−chelated salts had a significant positive impact on the bioaugmentation of Se and Zn in tea plants, which is consistent with previous results showing that nutrients in the form of amino chelates are efficiently absorbed and utilized [48,49]. In this study, foliar and soil application of glycine−chelated salts significantly increased the Se content of tea leaves. Foliar and soil application of Zn−Gly alone and the combination of Zn−Gly and Se−Gly significantly affected the Zn of tea leaves, in agreement with a previous report [50]. Foliar application of Se−Gly slightly elevated the Zn content of tea leaves. The improved Zn and Se absorption by crops following application of glycine−chelate salts may be attributed to the rapid conversion of glycine into ammonium, amide and aliphatic compounds, which affect the absorption of Zn and Se when they are used by plants as nitrogen sources [51]. In addition, plants absorb chelated nutrients more effectively than inorganic chlorides and sulfates, which may be due to the high membrane permeability of cells to amino acids [21]. The high water solubility of the chelated zinc and selenium complex also increases the utilization rate of zinc and selenium by plants [21]. When nutrient elements and chelating agents form stable chelated fertilizers, the utilization rate of nutrient elements is usually increased, leading to significant improvement in plant nutrient deficiency stress and quality [52]. It is easier for plants to form stable complexes containing amino acid−chelated compounds in comparison with inorganic salts [53]. As a result of this characteristic, various studies have shown that amino acid chelates have excellent effects on plant growth and productivity compared with simple inorganic chemical fertilizers [21]. In addition, chelated fertilizers have high nutrient stability and a slow nutrient release rate, which may more closely match the rate of absorption by the target crop, leading to more efficient use of nutrient elements in comparison with ordinary inorganic foliar fertilizer [54]. More importantly, the application of chelated fertilizer with a slow release rate is more likely to reduce toxicity on a local scale [55]. Therefore, amino acid−chelated fertilizers are suitable substitutes for chemical fertilizers in daily use [56].

### 3.3. Effects of Zn−Gly and Se−Gly on Functional Components of Tea Plant

*C. sinensis* has become one of the most popular plants in the human diet due to its beneficial effects on the human body, which mainly depend on the combined effects of various components such as TPs, AAs, L−theanine, and CAF [57,58]. Yan et al. found that the application of amino acid−chelated micronutrient fertilizer can also improve the appearance quality of fruit [59]. Cao et al. found that the combination of an organic base fertilizer and an amino acid−chelated microelement fertilizer significantly improved grape quality [60]. A follow−up study by Han et al. confirmed that appropriate addition of Mn−AA could improve the quality of Chinese cabbage and tomato [61]. In this study, foliar and soil application of Se−Gly alone significantly increased the content of TPs in tea leaves. TPs have been shown to effectively regulate the body’s innate immune response and enhance the response to novel coronavirus COVID-19 [24]. Application of Se−Gly or Zn−Gly alone reduced the accumulation of AAs, which is consistent with the results of previous studies [62]. A high ratio of non−ester catechins to ester catechins and a low bitterness index have been shown to improve the taste quality of tea infusions [63,64].

In the study, the non−esterified/esterified catechin ratio and catechin bitterness index have the potential to improve the taste of tea, which is consistent with the results of previous studies [63,64,65]. Foliar and soil application of the combination of Zn−Gly and Se−Gly increased the content of TPs, AAs, L−theanine, and total catechins in tea leaves, while it reduced the content of CAF, thus improving the quality of tea while maintaining a much lower level of environmental risk in comparison with commonly used inorganic salt fertilizers. Taken together, the results of this study demonstrate the optimal process parameters for efficient production of glycine−chelated forms of Zn and Se, as well as the positive effects of soil and foliar application of glycine−chelated Zn and Se on the nutritional quality of plants, in agreement with previous research results [30].

## 4. Materials and Methods

### 4.1. Synthesis of Zinc− and Selenium−Glycine Chelates

The physical and chemical properties, molecular structure, chelation strength, and chelation rate of the chelating agent together regulate the application effect of nutrients [54]. Glycine (Gly) was selected as the chelating agent in this study because it is an ideal choice for obtaining high−quality chelated products. The optimum industrial conditions of organic zinc glycine and organic selenium glycine were studied in the laboratory by water system synthesis [66]. The method parameters of Qin [67] were adopted with adjustment. The concentration of Gly was 4 %, 6 %, 8 %, or 10 %. The mass ratio was 1:1, 1:2, 2:1, or 3:1. The chelating temperature was 50, 60, 70, or 80 ℃. The pH was 4.0, 6.0, 8.0, or 9.0. The chelation time was 30, 40, 50, or 60 min. The detailed experimental design is shown in Table 4.

Zn−Gly were prepared according to the modified method of Chen Li et al. [68] to be adjusted. Among them, the concentration level of glycine is 2%,4%, 6%, 8%, and the mass ratio of gly and zinc sulfate to water. The average is 1:2, 1:1,2:1, 3:1, the chelating temperature level is 40, 60, 70, 80 °C, the pH level is 4.0, 6.0, 7.0, 8.0, and the chelating time level is 30, 60, 90, 120 min. Detailed design is shown in Table 5.

### 4.2. Characterization of Zn−Gly and Se−Gly Chelates

The Zn−Gly and Se−Gly sample was digested in an acidic solution and analyzed by ICP−OES (PQ9000, Jena Technology Co., Jena, Germany) according to the method of Yan et al. [69]. In order to determine the total amount chelated Zn and Se, 5 mL of each chelated filtrate was added to 10 mL of mixed acid (HNO_3_:HCIO_4_ at a ratio of 4:1) for digestion. After acid digestion, the Zn−Gly and Se−Gly solution was filtered through a 0.45 μm filter membrane and diluted to 25 mL with deionized water. Finally, the total amount of Zn and Se in the solution was determined by ICP−OES.

The Zn−Gly and Se−Gly chelation rates were calculated using the following equations:

Zinc chelation rate (%) = M1/M0 × 100(1)

Selenium chelation rate (%) = M2/M3 × 100(2)

where M0 and M1 are the total Zn and chelated Zn content, respectively, and M2 and M3 are the total Se and chelated Se content, respectively.

The structures of the Zn−Gly and Se−Gly samples with the highest chelation rates were analyzed. For ultraviolet spectroscopy, 0.1% Gly, Zn−Gly, and Se−Gly sample solution was scanned in the wavelength range of 200 to 500 nm by UV visible spectrophotometry (U−5100, Hitachi, Tokyo, Japan). For FT−IR spectroscopy, each Zn−Gly and Se−Gly sample was uniformly mixed with KBr (200 mg) in an agate mortar under infrared light [70]. FT−IR spectra were measured with a FT−IR JASCO (Model 6800 type A) spectrophotometer in the 4000 to 400 cm^−1^ range with a scanning speed of 2 mms^−1^ using a TGS detector. All infrared spectra of Gly, Zn−Gly and Se−Gly were collected using a Nexus FT−IR spectrometer (Nicolet 6800, Thermo Scientific, Waltham, MA, USA). Three biological replicates were tested for all treatments.

### 4.3. Pot Experiment

To test the efficacy of Zn−Gly and Se−Gly, a pot experiment was conducted with 1-year-old tea plants at the greenhouse of the College of Tea Science of Guizhou University. Zn and Se−deficient soil [71] was collected from the surface layer (depth of 0–20 cm) of tea plantations located at Guizhou University (26°11′ N–26°55′ N, 106°07′ E–107°17′ E) in Guizhou Province, China. Soil samples were completely air−dried at room temperature and homogenized. The samples were passed through a 5 mm sieve for the pot experiment, and through 0.25 and 0.149 mm sieves for physical and chemical analyses. Detailed characteristics of the collected soil samples are shown in Table 6.

The pot experiment was divided into 8 treatments. The experiment was designed are shown in Table 7, with 3 replicates per treatment. A randomized complete block design was used. Zn−Gly and Se−Gly were obtained from the optimally produced glycine−chelated zinc and glycine−chelated selenium from the process parameter experiments. One−year−old Fuding White tea plants (*C. sinensis* cv. Fuding−Dabaicha) were cultivated in mixed soil in 5 L plastic pots with bottom drainage. All pots were evenly placed in the greenhouse for environmental consistency. The light intensity was 200 mmol m^−2^s^−1^ and the photo/dark cycle was 14/10 h. The air temperature was maintained at 26/22 °C in the photo/dark periods, and the relative humidity was 70%. The soil was filled to a depth of 40 cm, with a diameter of 30 cm (approximately 10 kg dry weight soil per pot).

Tea seedlings were picked at 30, 60, 90, and 120 days after soil application of Zn−Gly and Se−Gly to check the material composition of the leaves. The picking standard was one bud and three leaves, and the fresh weight of each sample was about 5 g. Seedlings were picked at 10, 20, 30, and 60 days after foliar fertilizer application using the picking standard described above. The plant samples were washed three times with tap water, then washed three times with deionized water, and dried to constant weight at 80 °C. Finally, all samples were ground to pass through a 100−mesh sieve in a stainless−steel grinder and stored for Se/Zn content and quality component analysis. In the study, the potential toxic effects of Zn−Gly and Se−Gly application on plants and pests were not observed.

### 4.4. Determination of Chlorophyll Concentration

The concentrations of chlorophyll a, chlorophyll b, and carotenoids in tea leaves were determined spectrophotometrically at 470, 649, and 664 nm by Lichtenthaler, H.K. with a spectrophotometer (U−5100, Hitachi, Tokyo, Japan). Briefly, each 0.5 g fresh tea sample was extracted with 95% (10 mL) ethanol. The homogenized sample mixture was quantitatively transferred to a volumetric flask, bringing the volume to 25 mL and the mixture was filtered through filter paper. Calculation of chlorophyll and carotene concentrations was achieved using appropriate equations [72]:

Ch−a = 13.36A_664_ − 5.19A_649_; (3)

Ch−b = 27.43A_649_ − 8.12A_664_; (4)

C c = (1000A_470_ − 2.13 Ch−a − 87.63 Ch−b)/209; (5)

where A is absorbance, Ch−a is chlorophyll a, Ch−b is chlorophyll b, and C c is carotenoids.

### 4.5. Mineral Analysis

Plant samples were prepared and determined according to the Chinese National Standard (GB/T 27404-2008) [73]. Plant samples were digested with 4:1 (*v*/*v*) HNO_3_−HClO_4_ [74] using microwave-assisted acid digestion according to a previous study [75]. Briefly, each 0.2 g plant sample was precisely weighed in a 100 mL digestion tube with an additional 4:1 (HNO_3_−HClO_4)_. Next, 10 mL of combined HNO_3_−HclO_4_ was added to each plant sample. After acid digestion, the sample solutions were cooled and diluted with deionized water in the digestion tube. The total Zn and Se concentrations were determined using an ICP-OES (Optima 9000 DV) instrument [76]. Each sample was analyzed in triplicate.

### 4.6. Quality Parameters

The tea powder was oven-dried at 103 ± 2 °C to a constant weight to determine the dry matter content in each sample. The contents of tea polyphenols, catechins, and caffeine were measured according to the method in GB/T 8313-2018.22 according to the Chinese National Standard [77]. An Agilent 1260 Series U-HPLC Gradient System (Agilent Technologies, Santa Clara, CA, USA) equipped with a Zorbax Eclipse XDB-C18 column (250 mm × 4.6 mm, 5 μm) was used to separate, detect, and analyze the components of catechins. The free amino acid (AA) content was measured according to the method in GB/T 8314-2013 according to the Chinese National Standard [78]. Theanine was measured according to the method in GB/T 23193-2017 according to the Chinese National Standard [79]. All experiments were performed three times using independently prepared samples.

The catechin quality Index and ca”echi’ bitterness index were calculated according to the method of Liao et al. as follows [80]:

CAI = EGCG + EGC + ECG + GC/EC + C(6)

Non−esterified/esterified catechins = (EGC + C + GC + EC)/(EGCG + ECG + GCG + CG)(7)

### 4.7. Data Processing and Statistical Analysis

Microsoft EXCEL software was used for data statistics and processing. The data were subjected to an analysis of variance (ANOVA) using SPSS 18.0; error bars represent the mean ± standard deviation of data from three independent experiments. The analysis of variance was used to understand the differences between treatments, and the least significance difference (LSD) test at a 5% level of significance was used to compare the means. Figures were generated with Origin software 2021.

## 5. Conclusions

High quality Zn−Gly and Se−Gly products were prepared in this study. This study demonstrates that tea, as a functional food, has the potential to meet the nutritional shortcoming of Se and Zn that primary staple crops cannot meet by laying the foundation for the application of new amino acid−chelated Zn−Gly and Se−Gly fertilizers. Pot experiment results showed that Zn−Gly and Se−Gly can effectively increase the content of Zn and Se in tea leaves. Whether fertilizer was applied via foliar or soil application, application of Se−Gly alone or combined application of Zn−Gly and Se−Gly can achieve the “Se−enriched” standard, and application of Zn−Gly alone or combined application of Zn−Gly and Se−Gly can achieve the “Zn−enriched” standard. The application of Zn−Gly and Se−Gly prolongs the tea picking time, increases the economic value of tea, and provides an effective way for the human body to supplement Zn and Se. On the basis of Zn−Gly and Se−Gly effectively supplying Zn and Se to tea plants, the nutritional quality of tea and the functional components of tea plants can be improved in different ways; therefore, they are good substitutes for inorganic sodium selenite and zinc sulfate fertilizers. When using Zn−Gly and Se−Gly, the application concentration, application method, and the standard and time for picking fresh leaves should be applied by growers according to their own particular needs.

## Figures and Tables

**Table 1:** Orthogonal test (L_16_(4^5^)) of Gly−chelated Zn production parameters.

Number	A	B	C	D	E	Chelation Rate/%
1	1	1	1	1	1	55.88
2	1	2	2	2	2	56.65
3	1	3	3	3	3	58.68
4	1	4	4	4	4	55.49
5	2	1	2	3	4	61.06
6	2	2	1	4	3	75.80
7	2	3	4	1	2	62.34
8	2	4	3	2	1	68.78
9	3	1	3	4	2	68.29
10	3	2	4	3	1	66.23
11	3	3	1	2	4	65.92
12	3	4	2	1	3	66.86
13	4	1	4	2	3	62.43
14	4	2	3	1	4	68.72
15	4	3	2	4	1	70.45
16	4	4	1	3	2	66.92
K1	56.68	61.92	66.13	63.45	65.34	
K2	67.00	66.85	63.76	63.45	63.55	
K3	66.83	64.35	66.12	63.22	65.94	
K4	67.13	64.51	61.62	67.51	62.80	
R	10.46	4.93	4.51	4.28	3.14	

**Table 2:** Orthogonal test (L_16_(4^5^)) of Gly−chelated Se production parameters.

Number	A	B	C	D	E	Chelation Rate/%
1	1	1	1	1	1	38.98
2	1	2	2	2	2	39.16
3	1	3	3	3	3	40.21
4	1	4	4	4	4	39.02
5	2	1	2	3	4	45.37
6	2	2	1	4	3	50.75
7	2	3	4	1	2	54.89
8	2	4	3	2	1	56.75
9	3	1	3	4	2	50.24
10	3	2	4	3	1	51.23
11	3	3	1	2	4	50.89
12	3	4	2	1	3	52.02
13	4	1	4	2	3	45.73
14	4	2	3	1	4	49.89
15	4	3	2	4	1	46.26
16	4	4	1	3	2	47.28
K1	39.34	45.08	46.98	48.95	48.31	
K2	51.94	47.76	45.70	48.13	47.89	
K3	51.10	48.06	49.27	46.02	47.18	
K4	47.29	48.77	47.72	46.57	46.29	
R	12.60	3.69	3.57	2.92	2.01	

Note: K1, K2, K3, and K4 represent the chelation rate of the corresponding level 1, 2, 3, 4 under the five factors of pH, glycine concentration, glycine and ion ratio, chelation time, and chelation temperature; R represents the maximum value of K minus the minimum value under each factor.

**Table 3:** Characteristics of Gly−chelated Zn and Se.

Compound	Hygroscopicity	Solubility	pH	Color
Gly	Hygroscopic	Completely soluble	6.0	White
Zn−Gly	Hygroscopic	Completely soluble	6.2	White
Se−Gly	Hygroscopic	Completely soluble	6.7	Umber

**Table 4:** Orthogonal experimental factor level values for Se−Gly production.

Levels	Chelation pH (A)	Glycine Concentration (B)	Mass Ratio of Glycine to Sodium Selenite (C)	Chelation Time (D, min)	Chelation Temperature (E, °C)
1	4.0	4	1:1	30	50
2	6.0	6	1:2	40	60
3	8.0	8	2:1	50	70
4	9.0	10	3:1	60	80

**Table 5:** Orthogonal experimental factor level values for Zn−Gly production.

Levels	Chelation pH (A)	Glycine Concentration (B)	Mass Ratio of Glycine to Zinc Sulfate (C)	Chelation Time (D, min)	Chelation Temperature (E, °C)
1	4.0	2	1:2	30	40
2	6.0	4	1:1	60	60
3	7.0	6	2:1	90	70
4	8.0	8	3:1	120	80

**Table 6:** Soil properties from the soil experiment.

Soil Property	Experiment	Method
pH	5.4	pH meter (sample: 10 g soil in 100 mL deionized H_2_O)
Available nitrogen	45.49 mg/kg	Walkley–Black method
Available potassium	87.83 mg/kg	Colwell method
Available phosphorus	27.01 mg/kg	Colwell method
Available zinc	0.34 mg/kg	Lindsay and Norvell method
Available Selenium	1.24 µg/kg	Zhao and McGrath method

**Table 7:** Treatment design of Zn−Gly and Se−Gly (mg/L).

Name	Treatment	Detailed Information on Treatment
CK	Leaf control	Spray 100 mL of clean water
Zn−Gly	150 mg/L Zn	Spray 100 mL of Zn−Gly solution
Se−Gly	60 mg/L Se	Spray 100 mL of Se−Gly Solution
Zn−Gly + Se−Gly	60 mg/L Se + 150 mg/L Zn	Spray 100 mL mixed solution of Zn−Gly + Se−Gly Solution
CK	Soil control	Homogenized soil without Se and Zn addition
Zn−Gly	5 g/L Zn	Homogenized soil spiked with Zn−Gly Solution (500 mL)
Se−Gly	200 mg/L Se	Homogenized soil spiked with Se−Gly Solution (500 mL)
Zn−Gly + Se−Gly	200 mg/L Se + 5g/L Zn	Homogenized soil spiked with Zn−Gly + Se−Gly Solution (500 mL)

## Data Availability

Not applicable.
